# Effect of CdS loading on the properties and photocatalytic activity of MoS_2_ nanosheets

**DOI:** 10.1186/s13065-024-01250-y

**Published:** 2024-07-24

**Authors:** Ashmalina Rahman, Fazlurrahman Khan, James Robert Jennings, Ai Ling Tan, Young-Mog Kim, Mohammad Mansoob Khan

**Affiliations:** 1 Chemical Sciences, Faculty of Science, Universiti Brunei Darussalam, Jalan Tungku Link, Gadong, BE 1410 Brunei Darussalam; 2 Institute of Fisheries Science, Pukyong National University, Busan, 48513 Republic of Korea; 3 Marine Integrated Biomedical Technology Center, The National Key Research Institutes in Universities, Pukyong National University, Busan, 48513 Republic of Korea; 4 Research Center for Marine Integrated Bionics Technology, Pukyong National University, Busan, 48513 Republic of Korea; 5 Applied Physics, Faculty of Science, Universiti Brunei Darussalam, Jalan Tungku Link, Gadong, BE 1410 Brunei Darussalam; 6 Optoelectronic Device Research Group, Universiti Brunei Darussalam, Brunei Darussalam, Jalan Tungku Link, Gadong, BE 1410 Brunei Darussalam; 7 Department of Food Science and Technology, Pukyong National University, Busan, 48513 Republic of Korea

**Keywords:** MoS_2_, CdS, CdS@MoS_2_, Photocatalysis, Trapping agents, Pollutants

## Abstract

Molybdenum sulfide (MoS_2_) and modified MoS_2_ with different percentages of CdS (10%, 30%, and 50% CdS@MoS_2_) were successfully synthesized and characterized. The photocatalytic performance of the MoS_2_ and CdS@MoS_2_ was evaluated by degrading brilliant green (BG), methylene blue (MB), and rhodamine B (RhB) dyes under visible light irradiation. Amongst the synthesized photocatalysts, 50% CdS@MoS_2_ exhibited the highest photocatalytic activity, degrading 97.6%, 90.3%, and 75.5% of BG, MB, and RhB dyes, respectively within 5 h. The active species involved in the degradation processes were investigated. All trapping agents inhibited BG and MB degradation to a similar extent, indicating that all of the probed active species play an important role in the degradation of BG and MB. In contrast, h^+^ and O_2_^•−^ were found to be the main reactive species in the photocatalytic RhB degradation. A potential mechanism for the photocatalytic degradation of dyes using CdS@MoS_2_ has been proposed. This work highlights the potential of CdS@MoS_2_ as a photocatalyst for more efficient water remediation applications.

**Supplementary Information:**

The online version contains supplementary material available at 10.1186/s13065-024-01250-y.

## Introduction

Photocatalysis is a promising approach for the total mineralization of organic and inorganic pollutants because it can be performed under ambient conditions. It is also an energy-efficient, cost-effective, and simple method that does not produce secondary byproducts except CO_2_ and H_2_O [1, 2]. One of the widely used methods for the degradation of dyes and organic contaminants is the semiconductor-based photocatalysis process [3, 4]. This is because semiconductor-based materials have suitable band structures, and when the material is irradiated with an appropriate light source (where the photon energy is greater than the band gap energy of the semiconductor), electrons (e^−^) and positively charged holes (h^+^) are generated, which can produce superoxide (O_2_^•−^) radicals and hydroxyl (^•^OH) radicals, respectively [5, 6]. These reactive species are responsible for the degradation of organic dyes and other pollutants.

Semiconductors of the type MX_2_ are known as transition-metal dichalcogenides, where M is a transition metal and X is a chalcogen (S, Se, or Te) [7]. These chalcogenides have recently been recognized as potentially efficient photocatalysts, paving the way for the development of low-cost and earth-abundant photocatalysts. Molybdenum sulfide (MoS_2_) is a transition-metal dichalcogenide that has emerged as a remarkable photocatalyst for the photocatalytic degradation of pollutants among other chalcogenides [8, 9]. MoS_2_ exhibits good semiconducting properties and has a hexagonal structure similar to graphene with a narrow band gap in the range of 0.9–1.9 eV [10]. However, MoS_2_ exhibits an electron mobility of only 217 cm^2^ s^− 1^ V^− 1^, which increases the e^−^/h^+^ recombination rate when irradiated with light [11]. When MoS_2_ is irradiated by light with photon energies greater than or equal to the band gap energy, e^−^ are excited from the valance band (VB) to the conduction band (CB) and h^+^ are formed in the VB, as shown in equation (R1).

R1 $$\:{\text{M}\text{o}\text{S}}_{2}\left(\text{g}\text{r}\text{o}\text{u}\text{n}\text{d}\:\text{s}\text{t}\text{a}\text{t}\text{e}\right)\underrightarrow{\:h\nu\:\:}{\text{e}}_{\text{c}\text{b}}^{-}\left({\text{M}\text{o}\text{S}}_{2}\right)+{\text{h}}_{\text{v}\text{b}}^{+}\left({\text{M}\text{o}\text{S}}_{2}\right)$$

These e^−^/h^+^ may recombine (equation R2) or react with O_2_ (equation R3) and OH^−^ (equation R4) to produce O_2_^•−^ and ^•^OH radicals, which subsequently react with and degrade organic pollutants [12]. Such reactive species will not be generated if the e^−^/h^+^ recombines rapidly, and this will result in a reduction in the efficiency of the photocatalytic processes.

R2 $$\:{\text{e}}_{\text{c}\text{b}}^{-}\left({\text{M}\text{o}\text{S}}_{2}\right)+{\text{h}}_{\text{v}\text{b}}^{+}\left({\text{M}\text{o}\text{S}}_{2}\right)\to{\text{M}\text{o}\text{S}}_{2}\left(\text{g}\text{r}\text{o}\text{u}\text{n}\text{d}\:\text{s}\text{t}\text{a}\text{t}\text{e}\right)$$

R3 $$\:{\text{e}}_{\text{c}\text{b}}^{-}+{\text{O}}_{2\left(\text{a}\text{d}\text{s}\right)}\to{{\text{O}}_{2}}^{\bullet\:-}$$

R4 $$\:{\text{h}}_{\text{v}\text{b}}^{+}+{\text{O}{\text{H}}^{-}}_{\left(\text{a}\text{d}\text{s}\right)}\to{.}^{{\bullet}}{\text{OH}}$$

Fast e^−^/h^+^ recombination of MoS_2_ can be hindered by many approaches such as decoration with metals, non-metal doping, metal doping, and coupling with other semiconductors to form composites, which have been widely and intensively attempted in recent years [1, 13–15]. The fabrication of composite heterostructures with other semiconductors is one of the most effective ways to improve the separation of photogenerated charge carriers and hinder the recombination of these carriers. The appropriate combination of MoS_2_ with other semiconductors to fabricate heterostructures can significantly enhance the separation of photogenerated carriers and enhance its photocatalytic performance. Various MoS_2_-based heterostructures such as MoS_2_/WS_2_ [16], MoS_2_/ZnS [17] MoS_2_/ZnO [18] and MoS_2_/g-C_3_N_4_ [19] have been fabricated as promising materials for photocatalytic reactions. Among these combinations, cadmium sulfide (CdS) has recently received considerable interest owing to its suitable direct band gap of 2.4 eV [20] which can be excited by visible photons to generate charge carriers. CdS has been extensively used in wastewater treatment [21] organic dye degradation [22] water splitting [23, 24] and CO_2_ reduction [25]. However, pristine CdS is unable to satisfy the requirements of practical applications due to photocorrosion and the rapid recombination of photogenerated charge carriers. Fortunately, these limitations can be partly overcome by combining CdS with MoS_2_, resulting in a material with substantially improved photocatalytic efficiency.

In this study, the photocatalytic activity of CdS@MoS_2_ composite heterostructure synthesized via a hydrothermal method was investigated. The effect of CdS on the structural and optical properties of the obtained MoS_2_ was characterized using X-ray diffraction, X-ray photoelectron spectroscopy, Fourier transform infrared spectroscopy, Raman spectroscopy, UV-visible diffuse reflectance spectroscopy, photoluminescence spectroscopy, transmission electron microscopy, and Brunauer-Emmett-Teller surface area analysis. By combining MoS_2_ with CdS, the recombination rate of photogenerated carriers was significantly reduced and an enhancement in the photocatalytic degradation of three different dyes with a low photocatalyst dosage was achieved. A further in-depth study of the active species responsible for the dye degradation was also performed, providing insights into the underlying degradation mechanisms.

## Experimental section

### Chemicals

All reagents were used without further purification. For the synthesis, ammonium heptamolybdate ((NH_4_)_6_Mo_7_O_24_, 98%) as the Mo source was purchased from BDH Chemical Ltd, UK. Cadmium sulfate (CdSO_4_·8/3H_2_O, 98%) as the Cd source, and thiourea (NH_2_CSNH_2_, ≥ 99%) as the sulfur (S) source were purchased from Merck, Germany. For the photocatalytic application tests, methylene blue (MB) was obtained from Merck, while rhodamine B (RhB) and brilliant green (BG) were obtained from Sigma-Aldrich. The distilled water was purified using Aquatron, England, and ethanol was purchased from Duksan Pure Chemicals Co. Ltd, South Korea. For the active species trapping experiments, isopropanol was purchased from Merck, benzoquinone was from Acros, and ethylenediaminetetraacetic acid disodium salt dihydrate (EDTA-2Na) was purchased from Fluka.

### Instrumentation

Powder X-ray diffraction (XRD, MiniflexII; Rigaku, Japan) was used to examine the crystal structure and phase purity of the synthesized materials with step size of 0.0262° in the range of 10 to 80°. The determination of the chemical states and elemental compositions of the synthesized materials was carried out using X-ray photoelectron spectroscopy (XPS, Kratos Analytical, AXIS Nova). The XPS analytical chamber was pumped down to ultra-high vacuum (4.6 × 10^− 9^ Torr) and energies were referenced to the adventitious carbon C1s peak at 284.8 eV. The vibrational modes present in the synthesized materials were identified using Fourier transform infrared spectroscopy (FT-IR) via the KBr pellet method. The FT-IR spectra of these materials were recorded using an IRspirit FTIR spectrometer (Shimadzu, Japan) in the range of 400–4000 cm^− 1^. Raman spectra were obtained using a Micro Raman Spectrometer (JASCO NRS-5100, Japan) equipped with a 532.06 nm laser. The optical band gap of the materials was examined using Ultraviolet-visible diffuse reflectance spectroscopy (UV-visible DRS, Shimadzu UV-2600i, Japan). A photoluminescence (PL) study was carried out using an F-7000 Fluorescence spectrophotometer (Hitachi High Tech, Japan) with an excitation wavelength of 400 nm. The morphology and crystallographic information of the synthesized materials were analyzed using field-emission transmission electron microscopy (FE-TEM) with an acceleration voltage of 200 kV and selected area electron diffraction (SAED) conducted with JEM-F200 (JEOL Ltd., Tokyo, Japan). The surface area analysis of MoS_2_ and 50% CdS@MoS_2_ was measured by a surface area analyzer (Quantachrome autosorb-iQ, Austria). For photocatalytic studies of the synthesized materials, the experiments were carried out in a Toption (TOPT-V) photochemical reactor with a 300 W Xe lamp as the light source (wavelength > 350 nm). The intensity of the light at the location of the reaction vessel was ~ 14 mW/cm^2^, as measured by a Thorlabs PM100D power meter with a S401C thermal sensor. The distance between the reaction vessels and the water jacket surrounding the Xe lamp was 5 cm. A Shimadzu UV-1900 UV–visible spectrophotometer was utilized to monitor the absorbance of BG, MB, and RhB dyes over a wavelength range of 200–800 nm.

### Preparation of MoS_2_

MoS_2_ was prepared using a hydrothermal method. In a typical synthesis, 0.3309 g of (NH_4_)_6_Mo_7_O_24_ and 0.5707 g of thiourea were added into a polytetrafluoroethylene (PTFE) container filled with 20 mL of distilled water. The mixture was then purged with N_2_ gas, sonicated for 5 min, and sealed in a stainless steel autoclave. The autoclave was then heated at 200 °C for 6 h and later cooled to room temperature. The black powder product was collected by centrifugation and washed with distilled water and ethanol three times. Finally, the obtained product was dried at 60 °C for 4 h.

### Preparation of CdS

CdS was synthesized using a hydrothermal method. Typically, 0.1142 g of thiourea and 0.3848 g of CdSO_4_·8/3H_2_O were dissolved in 20 mL of distilled water. The reaction solution was purged with N_2_ gas and sonicated for 5 min in the PTFE container. The PTFE container was then sealed in the stainless-steel autoclave and heated at 200 °C for 6 h. After that, it was cooled to room temperature, and a bright yellow product was collected by centrifugation. The product was washed three times with distilled water and ethanol. Finally, it was dried at 60 °C in a drying oven.

### Preparation of CdS@MoS_2_ composites

For the synthesis of CdS@MoS_2_ composites, a well-dispersed and stable black suspension was prepared by sonicating 0.2 g of the synthesized MoS_2_ powder in a PTFE container filled with 20 mL of H_2_O. To investigate the effect of CdS loading amount on photocatalyst performance, different weight percentages of CdS from 10 to 50% were used. Appropriate amounts of CdSO_4_·8/3H_2_O and thiourea were added into the PTFE container. The mixture was then purged with N_2_ gas, sonicated for 5 min, and sealed in a stainless steel autoclave. The mixture was reacted hydrothermally at 200 °C for 6 h to obtain a series of composites. The reaction mixture was then cooled to room temperature and the collected CdS@MoS_2_ composites were washed with distilled water and ethanol three times. The obtained CdS@MoS_2_ samples were labeled as 10% CdS@MoS_2_, 30% CdS@MoS_2_, and 50% CdS@MoS_2_.

### Photocatalytic degradation of dyes

The photocatalytic activity of the pristine MoS_2_, CdS, 10% CdS@MoS_2_, 30% CdS@MoS_2_, and 50% CdS@MoS_2_ was evaluated by degrading different dyes (BG, MB, and RhB) at room temperature under visible light irradiation. A typical process was carried out as follows: 5 mg of synthesized materials were sonicated in 50 mL of 10 ppm BG, MB, or RhB aqueous solution. Then, the suspension was kept stirred in the dark for 3 min to achieve the adsorption-desorption equilibrium of dye on the surface of the synthesized materials before exposing it to visible light. Approximately 3 mL suspension was transferred into a centrifuge tube every 1 h to separate the photocatalysts and the dye pollutant. The clear aliquots were analyzed using a UV–visible spectrophotometer in the range of 200 to 800 nm. The photocatalytic activity of synthesized pristine MoS_2_, CdS, 10% CdS@MoS_2_, 30% CdS@MoS_2_, and 50% CdS@MoS_2_ was estimated by measuring the percentage of dyes degradation using the following relation (1):

1 $$\:\text{\%}\:\text{d}\text{e}\text{g}\text{r}\text{a}\text{d}\text{a}\text{t}\text{i}\text{o}\text{n}=\frac{{A}_{0}-{A}_{t}}{{A}_{0}}\times\:100$$

where *A*_0_ denotes the initial absorbance (the absorbance at time *t* = 0 h) and *A*_*t*_ denotes the absorbance of aqueous dye solution after time *t* of treatment. Both *A*_0_ and *A*_*t*_ are recorded at the absorbance maximum (*λ*_max_) of the dye. The *λ*_max_ of BG, MB, and RhB, is 620, 658, and 553 nm, respectively.

### Active species trapping experiments

To further investigate the main reactive species responsible for the photocatalytic degradation of dyes using 50% CdS@MoS_2_ composite, trapping experiments were carried out in the presence of three typical active species trapping agents: isopropanol, benzoquinone, and EDTA-2Na, which are utilized as scavengers of ^•^OH, O_2_^•−^, and h^+^, respectively. These trapping agents were added separately to the aqueous dye solution at the beginning of the photocatalytic reaction. This experiment was carried out under the same conditions as described in sub-heading “Photocatalytic degradation of dyes”.

## Results and discussions

### Powder X-ray diffraction analysis

**Fig. 1:**
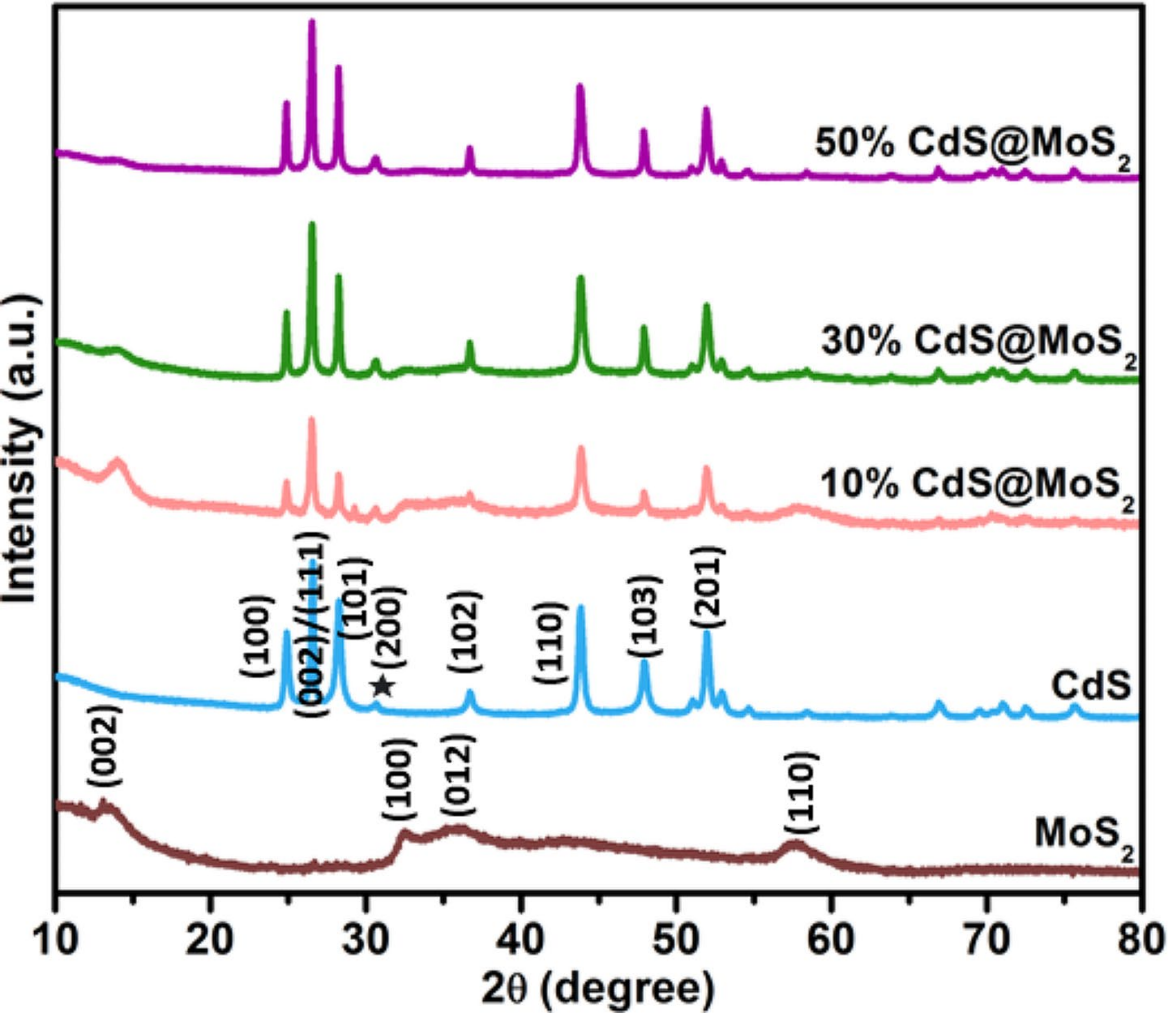
XRD patterns of MoS_2_, CdS, 10% CdS@MoS_2_, 30% CdS@MoS_2,_ and 50% CdS@MoS_2_

XRD was employed to study the purity, phase, and crystal structure of the synthesized materials. The XRD patterns of pristine MoS_2_, CdS, 10% CdS@MoS_2_, 30% CdS@MoS_2_, and 50% CdS@MoS_2_ are shown in Fig. 1. Pristine MoS_2_ exhibited peaks at 2θ values of 13.9°, 32.4°, 35.7°, and 57.8°, corresponding to the (002), (100), (012), and (110) planes of 2 H/3R-MoS_2_ (as shown in Figure S1(a)), suggesting the formation of a mixed phase with respect to the standard diffraction peaks found in JCPDS 37-1492 [26] and JCPDS 17–0744 [27]. The “2H” and “3R” prefixes indicate hexagonal and rhombohedral symmetries, respectively and the number represents the number of layers in the unit cell. The diffraction peaks of MoS_2_ are quite broad and low in intensity, indicating that the crystallite size is very small and has relatively poor crystallinity [28, 29]. The diffraction peaks of pristine CdS shown in Fig. 1 could be indexed to hexagonal (JCPDS 65-3414) and cubic CdS (JCPDS 65-2887). The distinct diffraction peaks at 2θ values of 24.9°, 26.6°, 28.3°, 36.6°, 43.8°, 47.9°, and 51.9° corresponds to the (100), (002), (101), (102), (110), (103), and (201) planes were attributed to hexagonal CdS. While peaks of (111) and (200) at 2θ values of 26.6 and 30.7 correspond to the cubic CdS as shown in Figure S1(b). The diffraction patterns of the CdS@MoS_2_ composites exhibited peaks characteristic of MoS_2_ and CdS. As the loading amount of CdS increases from 10 to 50%, it is observed that the diffraction peaks of CdS become sharper and the diffraction peaks of MoS_2_ become weaker. Moreover, for 50% CdS@MoS_2_, additional diffraction peaks located at 24.6°, 26.5°, 28.3°, 36.7°, 43.8°, 47.9°, and 51.9° are observed, which can be ascribed to the (100), (002), (101), (102), (110), (103), and (201) planes of hexagonal wurtzite CdS [30]. The overlapped peak centered at 26.5° and the presence of a weak peak at 30.7° can be assigned as the (111) and (200) planes of cubic CdS, respectively, which implies that the obtained 50% CdS@MoS_2_ material also consisted of a mixed phase of hexagonal and cubic CdS [31]. These results indicate that CdS was successfully loaded onto the surface of MoS_2_.

### X-ray photoelectron spectroscopy

**Fig. 2:**
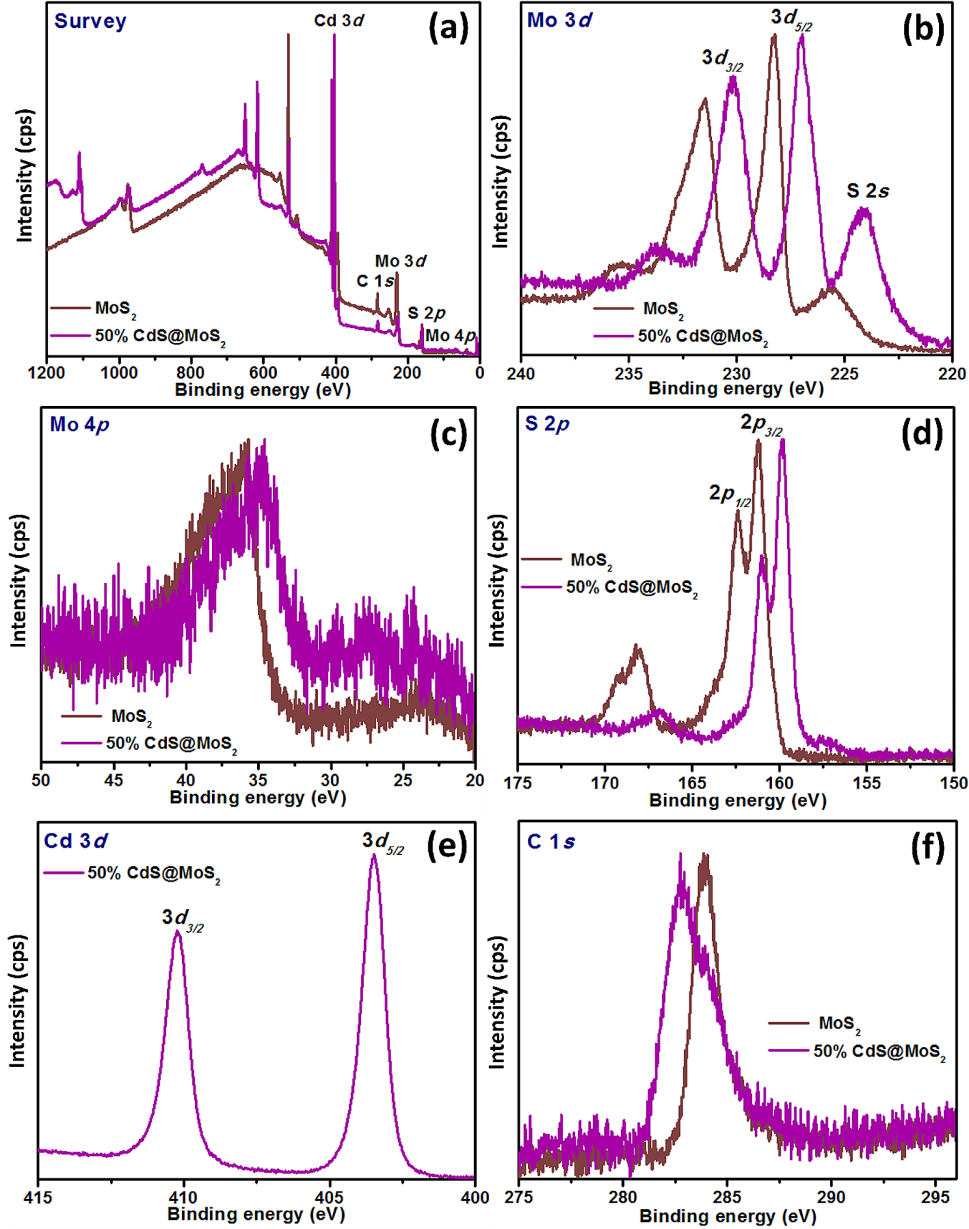
XPS spectra of MoS_2_ and 50% CdS@MoS_2_: (**a**) survey scan, (**b**) Mo 3*d*, (**c**) Mo 4*p*, (**d**) S 2*p*, (**e**) Cd 3*d*, and (**f**) C 1*s*

The oxidation states and elemental composition of MoS_2_ and 50% CdS@MoS_2_ were determined using XPS. In Fig. 2(a), the XPS survey reveals the presence of Mo, S, and adventitious C elements for pristine MoS_2,_ and the same elements were also observed in the 50% CdS@MoS_2_ with the addition of Cd indicating the co-existence of MoS_2_ and CdS in the composite [32]. These results corroborate the high purity of all samples corresponding to their XRD. For pristine MoS_2_, the characteristic peaks for Mo 3*d*_3/2_ and Mo 3*d*_5/2_ were visible as a doublet at 2315 eV and 228.3 eV which indicated the presence of Mo^4+^. Moreover, the Mo 3*d* spectra of 50% CdS@MoS_2_ display two peaks at 230.2 and 227.0 eV assigning to Mo^4+^ 3*d*_3/2_ and Mo^4+^ 3*d*_5/2_. It can also be observed that the peaks are shifting to lower binding energy positions (Fig. 2(b)). Moreover, the peak centered at 224 eV ascribed to S 2*s* is also observed [33]. The binding energy shifts reflect changes in the electronic structures of the as-synthesized materials, further showing the strong interaction between MoS_2_ and CdS [30, 34–36]. As shown in Fig. 2(c) the peak located at 37.1 and 35.1 eV corresponds to the Mo 4*p* peak of MoS_2_ and 50% CdS@MoS_2_, respectively.

The spectrum of S 2*p* of pristine MoS_2_ (Fig. 2(d)) indicates that the peak can be deconvoluted into two peaks, one located at 162.4 and the other at 161.2 eV with an energy difference of 1.2 eV. Meanwhile, the binding energies of S 2*p* of 50% CdS@MoS_2_ are located at 161.0 and 159.8 eV with the same energy difference of 1.2 eV. These peaks can be assigned to the typical S 2*p*_1/2_ peaks and S 2*p*_3/2_ of S^2−^ in the as-synthesized materials. As seen in Fig. 2(e) of 50% CdS@MoS_2_, two characteristic peaks centered at the binding energy of 410.2 and 403.5 eV correspond to Cd 3*d*_5/2_ and Cd 3*d*_3/2_, respectively. The binding energy values and the splitting energy of 6.75 eV are consistent with the reported results of Cd^2+^ in CdS. The typical C 1*s* spectra shown in Fig. 2(f) were derived from the XPS instrument itself. Thus, the above XPS results further confirmed the formation of the CdS@MoS_2_ composite.

### Fourier transform infrared spectroscopy

**Fig. 3:**
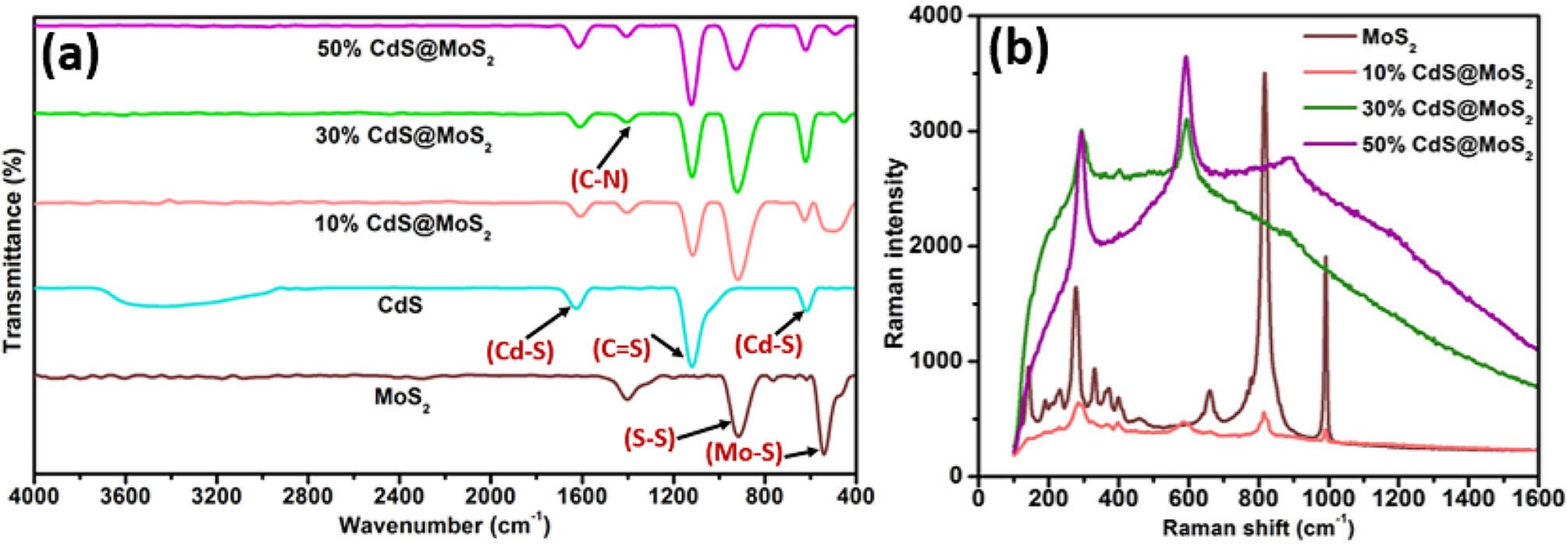
(**a**) FT-IR spectra and (**b**) Raman spectra of MoS_2_, CdS, 10% CdS@MoS_2_, 30% CdS@MoS_2_, and 50% CdS@MoS_2_

The presence of different vibrational modes of the synthesized materials was examined by FT-IR spectroscopy. Figure 3(a) shows the FT-IR spectra of MoS_2_, CdS, 10% CdS@MoS_2_, 30% CdS@MoS_2_ and 50% CdS@MoS_2_. The FTIR of MoS_2_ showed that the bands at 463 and 913 cm^− 1^ can be ascribed to the Mo-S and S-S bonds, respectively [18, 37, 38]. The band centered around 1390 cm^− 1^ can be ascribed to the C-N vibration of thiourea [39]. The bands located at 622, 1128, 1401, and 1633 cm^− 1^ can be attributed to the characteristic IR absorption of the Cd–S bond, confirming the formation of CdS [1, 32]. For the FT-IR spectrum of CdS, the broad band centered at 3307 cm^− 1^ can be assigned to the O–H stretching vibration of the adsorbed H_2_O from the atmosphere. A weak band at 463 cm^− 1^ corresponding to the Mo-S stretching vibration can also be observed in the spectra of all CdS@MoS_2_ composites. The FT-IR spectra of 10% CdS@MoS_2_, 30% CdS@MoS_2_, and 50% CdS@MoS_2_ exhibited similar Cd-S bands at approximately 925 and 1633 cm^− 1^. This confirms the presence of both MoS_2_ and CdS. Thus, FTIR also validates the successful loading of CdS onto MoS_2_.

### Raman spectroscopy

Raman spectroscopy was used to determine the presence of optical phonon vibrations in MoS_2_, CdS, 10% CdS@MoS_2_, 30% CdS@MoS_2_, and 50% CdS@MoS_2_. From Fig. 3(b), the two peaks observed at 372 and 402 cm^− 1^ were attributed to the E^1^_2g_ and A_1g_ modes of MoS_2_ [40]. The two identified phonon modes in MoS_2_ are E^1^_2g_ and A_1g_ which correspond to in-plane opposite vibrations of molybdenum/sulfur atoms and out-of-plane vibrations of sulfur atoms, respectively [41]. Between the two noticeable peaks, there is a 30 cm^− 1^ difference in the frequency [42]. In Figure S2, pristine CdS exhibited two characteristic peaks at 292 and 589 cm^− 1^, corresponding to the hexagonal phase of CdS [33]. Moreover, the Raman active MoS_2_ peak at 403 cm^− 1^ with low intensity was also observed in 10% CdS@MoS_2_, 30% CdS@MoS_2_, and 50% CdS@MoS_2_ composites. There were two additional Raman active peaks centered at 292 and 589 cm^− 1^ observed in 10% CdS@MoS_2_, 30% CdS@MoS_2_, and 50% CdS@MoS_2_ which could be attributed to the longitudinal optical (LO) mode of the hexagonal CdS, 1LO and 2LO respectively. The Raman spectra of the CdS@MoS_2_ composites showed the characteristic features of both MoS_2_ and CdS. Thus, these results also confirm the successful formation of CdS, MoS_2_, and CdS@MoS_2_ composites.

### UV-visible diffuse reflectance spectroscopy

**Fig. 4:**
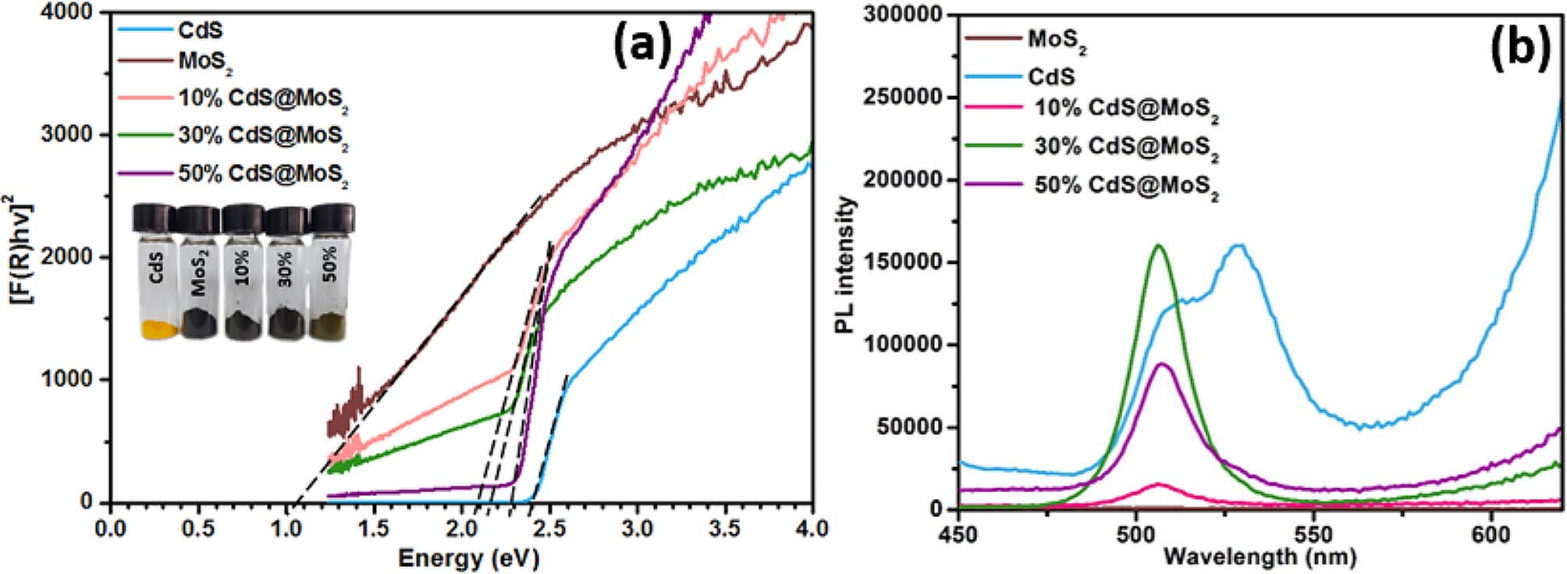
(**a**) Tauc plots constructed from Kubelka–Munk transformed diffuse reflectance spectra and (**b**) PL spectra of MoS_2_, CdS, 10% CdS@MoS_2_, 30% CdS@MoS_2_, and 50% CdS@MoS_2_

UV-vis DRS spectroscopy was employed to determine the optical properties of pristine MoS_2_, CdS, and CdS@MoS_2_ composites with different loading amounts of CdS (from 10 to 50%). Due to their black nature, the pristine MoS_2_ exhibited a broad absorption peak instead of a sharp absorption peak in the UV-visible region. Liu et al. reported similar absorption spectra of MoS_2_ [20]. For pristine CdS, the absorption spectrum has a clear onset around 520 nm, corresponding to a band gap energy of 2.4 eV. In contrast, pristine MoS_2_ exhibited a narrow band gap energy of 1.03 eV. Figure S3 shows absorbance (computed as the negative logarithm of the reflectance) spectra of pristine MoS_2_, CdS, 10% CdS@MoS_2_, 30% CdS@MoS_2_, and 50% CdS@MoS_2_. The difference in the absorption properties of the as-synthesized materials is also reflected in the change of their color (see the inset of Fig. 4).

The effective band gaps of 10% CdS@MoS_2_, 30% CdS@MoS_2_, and 50% CdS@MoS_2_ were calculated by constructing Tauc plots from Kubelka–Munk transformed diffuse reflectance data, as shown in Fig. 4(a). The direct band gaps of 10% CdS@MoS_2_, 30% CdS@MoS_2_, and 50% CdS@MoS_2_ were estimated to be 2.0, 2.2, and 2.3 eV, respectively. As the CdS content increases, the band gap energy of CdS@MoS_2_ gradually increases from 2.0 eV to 2.3 eV. To investigate the ability of these materials to harvest visible light energy (due to their narrow band gaps) and effectively utilize the photogenerated charge carriers, the photocatalytic degradation of various dyes was studied, and the results were presented in Table 1 and discussed in Sect. “Photocatalytic degradation of dyes”.

### Photoluminescence spectroscopy

PL spectra were acquired to study the recombination and interfacial transfer of charge carriers in the synthesized materials. The PL spectra (excitation wavelength 400 nm) of MoS_2_, CdS, 10% CdS@MoS_2_, 30% CdS@MoS_2_, and 50% CdS@MoS_2_ are shown in Fig. 4(b). The pristine CdS exhibits two PL peaks at 510 and 528 nm, while the pristine MoS_2_ exhibits no detectable PL peaks over the same wavelength range. In contrast to pristine CdS, the spectra of the CdS@MoS_2_ composites only exhibit a single emission peak at 506 nm. The strong quenching of the 528 nm PL peak in the composites indicates reduced recombination in the CdS, most likely due to charge transfer from CdS to MoS_2_. Compared to other composites, 10% CdS@MoS_2_ exhibited the lowest PL intensity possibly due to the least amount of CdS loaded onto the MoS_2_ surface. Whereas 30% CdS@MoS_2_ showed the highest PL intensity which generally reflects rapid recombination of charge carriers and usually results in lower photocatalytic activity. Moreover, the 50% CdS@MoS_2_ displayed a reduced PL intensity compared to 30% CdS@MoS_2_, indicating the effective separation of photo-induced charge carriers at the CdS/MoS_2_ interface.

### Transmission electron microscopy

**Fig. 5:**
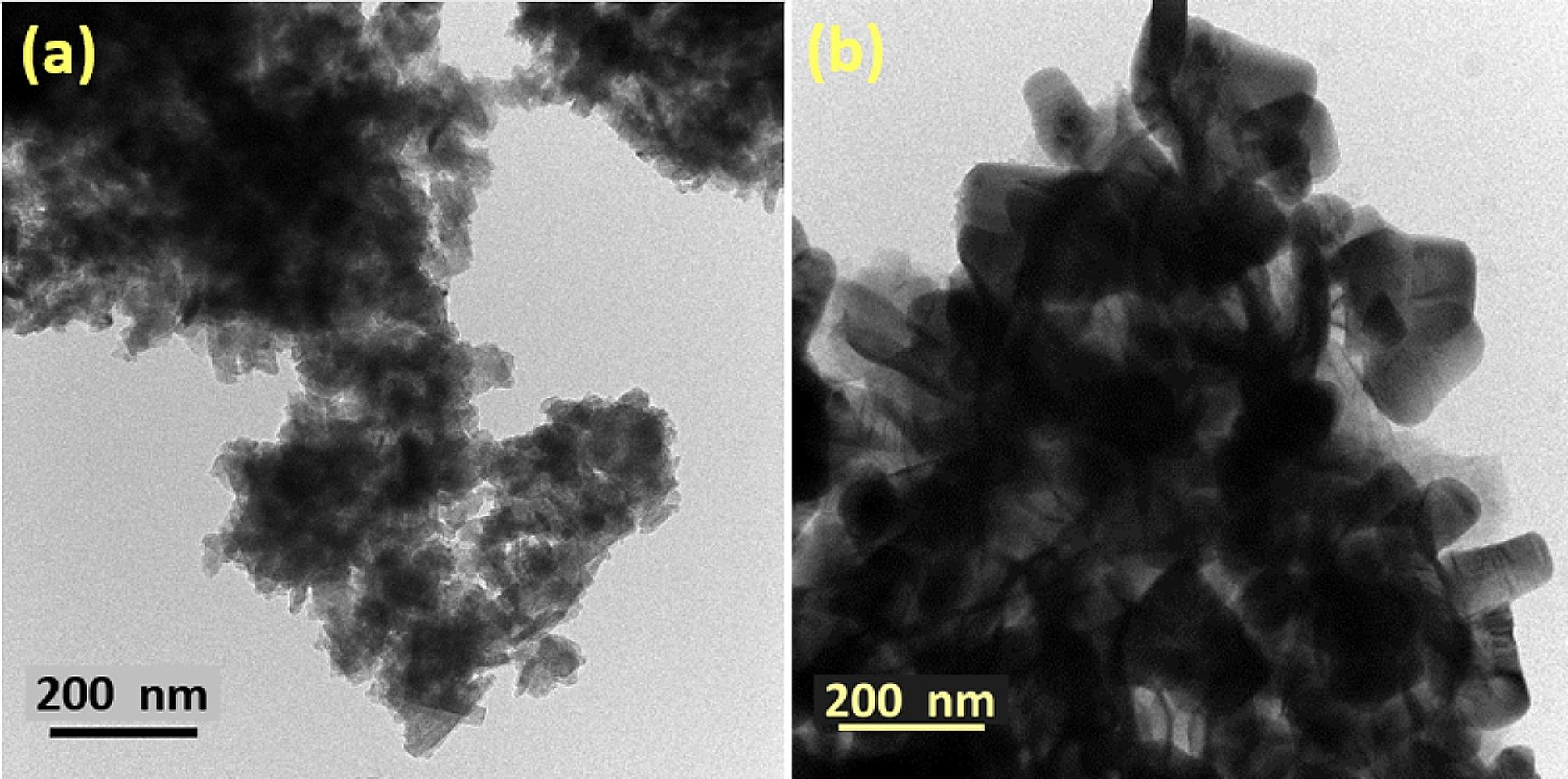
TEM images of (**a**) MoS_2_ and (**b**) 50% CdS@MoS_2_ composite

The morphologies of the as-synthesized pristine MoS_2_ and 50% CdS@MoS_2_ were investigated by TEM, as shown in Fig. 5. As can be seen in Fig. 5(a), the TEM image of pristine MoS_2_ consists of thin nanosheets. After the modification of MoS_2_ with 50% CdS, the morphology remained mostly unchanged with some agglomeration. In addition, HR-TEM was also used to further explore the nanostructures of MoS_2_ and 50% CdS@MoS_2_. In Figure S4(a), the interplanar spacing of 0.27 nm observed in the MoS_2_ high-resolution TEM (HR-TEM) could be ascribed to the (101) plane of 3R-MoS_2_ and (100) plane of 2 H-MoS_2_ [43, 44]. While the HR-TEM of 50% CdS@MoS_2_, as shown in Figure S4(b), revealed the interplanar spacing of 0.32 nm could be assigned to (101) plane of hexagonal CdS and 0.34 nm could be assigned to (111) plane of cubic CdS. Additionally, the presence of 0.27 nm spacing in the HR-TEM of 50% CdS@MoS_2_ confirms the successful formation of CdS@MoS_2_ composites. The selected area electron diffraction (SAED) shown in Figure S5 and Figure S6 confirms the polycrystalline nature of the MoS_2_ and 50% CdS@MoS_2_. Combined with the XRD and XPS characterization, these results confirm the formation of and close contact between CdS and MoS_2_, which is advantageous for interfacial charge separation.

### Brunauer-Emmett-Teller surface area analysis

The surface area of MoS_2_ and 50% CdS@MoS_2_ was analyzed from N_2_ adsorption/desorption isotherm as shown in Figure S7 and the corresponding multipoint BET plot (inset graph in Figure S7). The adsorption/desorption isotherms of the synthesized materials are the characteristic IV curves with prominent H3-type hysteresis loops, illustrating the synthesized materials exhibited porous structures [45]. The MoS_2_ exhibited a surface area of 55.421 m^2^/g. With the introduction of 50% CdS onto MoS_2_, the surface area increases to 63.542 m^2^/g. This can reasonably explain why the formation of CdS@MoS_2_ composite is beneficial in increasing the surface area and the number of active sites.

## Photocatalytic degradation of dyes

The photocatalytic activity of pristine CdS, MoS_2_, and CdS@MoS_2_ composites with various CdS percentages (10, 30, and 50%) was investigated by monitoring the degradation of BG, MB, and RhB under visible light irradiation. In order to study the stability of the dyes, the dye solutions were first subjected to a photolysis process. This was also carried out as a positive control experiment. The results show that only 4%, 3%, and 6% degradation were observed in aqueous BG, RhB, and MB blank dyes (in the absence of photocatalysts). This means that the photocatalyst is required for the acceleration of the photocatalytic reaction.

**Fig. 6:**
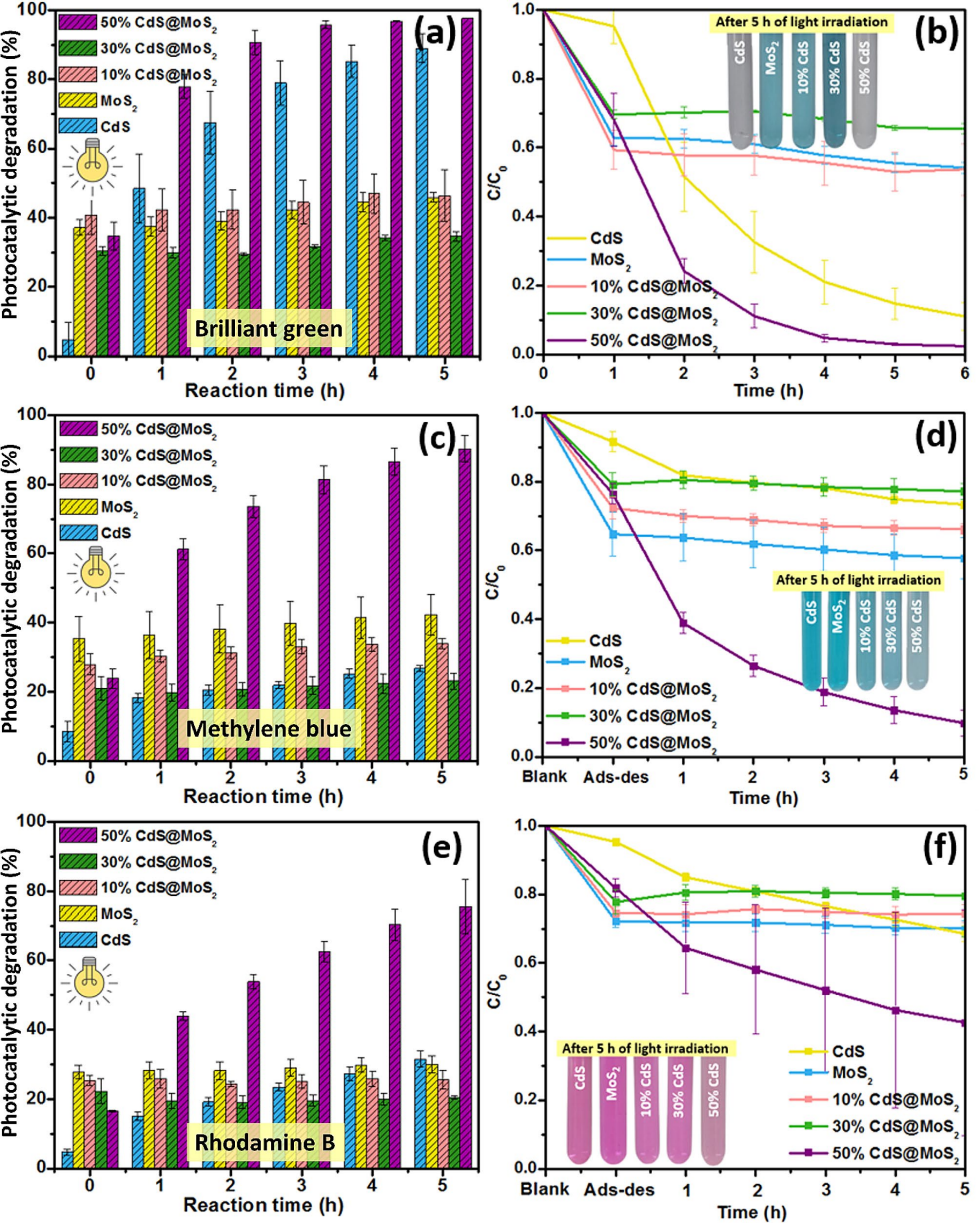
Photocatalytic efficiency and *C*/*C*_0_ plots of MoS_2_, CdS, 10% CdS@MoS_2_, 30% CdS@MoS_2_, and 50% CdS@MoS_2_ for (**a**,** b**) BG (**c**,** d**) MB, and (**e**,** f**) RhB degradation under visible light irradiation, respectively

From the photocatalytic degradation experiment, it was found that pristine CdS degraded about 88.9% ± 4.13% of BG within 5 h while pristine MoS_2_ showed high adsorption and poor photocatalytic activity (45.9% ± 1.50%) as shown in Fig. 6(a). Using different percentages of CdS, the photocatalytic performance of MoS_2_ was remarkably enhanced. The average degradation percentages were found to be 46.3% ± 7.46%, 34.6% ± 1.50%, and 97.6% ± 1.74% for 10% CdS@MoS_2_, 30% CdS@MoS_2_ and 50% CdS@MoS_2_, respectively. The photocatalytic degradation of MB in aqueous solution under visible light irradiation using MoS_2_, CdS, 10% CdS@MoS_2_, 30% CdS@MoS_2_, and 50% CdS@MoS_2_ were shown in Fig. 6(c). Both pristine CdS and MoS_2_ showed poor photocatalytic activity with only 26.7% ± 0.85% and 42.3% ± 5.96% degradation of MB, respectively. The photocatalytic performance of MoS_2_ was remarkably enhanced as the amount of CdS loading increased from 10 to 50% and the average degradation percentages were about 33.9% ± 1.42%, 23.0% ± 2.26%, and 90.3% ± 3.79% for 10% CdS@MoS_2_, 30% CdS@MoS_2_, and 50% CdS@MoS_2_, respectively. The photocatalytic activities of MoS_2_, CdS, 10% CdS@MoS_2_, 30% CdS@MoS_2_, and 50% CdS@MoS_2_ were also evaluated by degrading RhB in aqueous solution under visible light irradiation as shown in Fig. 6(e). Both pristine CdS and MoS_2_ showed poor photocatalytic activity with only 31.5% ± 2.29%, and 30.0% ± 2.37% degradation of RhB, respectively. Using different percentages of CdS, the photocatalytic degradation performance of MoS_2_ was remarkably enhanced and the average photocatalytic degradation percentages were about 25.6% ± 2.65%, 20.5% ± 0.53%, and 75.5% ± 7.78% for 10% CdS@MoS_2_, 30% CdS@MoS_2_, and 50% CdS@MoS_2_, respectively.

**Table 1:** The effective band gap energy and photocatalytic degradation efficiency of MoS_2_, CdS, 10% CdS@MoS_2_, 30% CdS@MoS_2_, and 50% CdS@MoS_2_

Synthesized materials	Band gap energy (eV)	Photocatalytic degradation (%)		
		Brilliant green	Methylene blue	Rhodamine B
MoS_2_	1.03	45.9 ± 1.50	42.3 ± 5.96	30.0 ± 2.37
CdS	2.40	88.9 ± 4.13	26.7 ± 0.85	31.5 ± 2.29
10% CdS@MoS_2_	2.00	46.3 ± 7.46	33.9 ± 1.42	25.6 ± 2.65
30% CdS@MoS_2_	2.20	34.6 ± 1.50	23.0 ± 2.26	20.5 ± 0.53
50% CdS@MoS_2_	2.30	97.6 ± 1.74	90.3 ± 3.79	75.5 ± 7.78

Based on these results, it can be observed that the photocatalytic activity of MoS_2_ increases and then decreases when the amount of CdS loaded increases from 10 to 30% for BG, MB, and RhB dyes. This is owing to an inadequate amount of CdS being loaded onto MoS_2_ in both 10% and 30% CdS@MoS_2_ composites, indicating the importance of CdS in the composites. On the other hand, 50% CdS@MoS_2_ exhibited the highest photocatalytic efficiency against BG, MB, and RhB dyes compared to the other synthesized materials. This may be ascribed to the high loading amount of CdS on the MoS_2_ surface. Figure 6(b, d, and f) present *C*/*C*_0_ vs. time graphs that provide a clearer understanding of the effect of CdS loading on the photocatalytic performance of MoS_2_ for the degradation of BG, MB, and RhB dyes, respectively. The *C*/*C*_0_ metric is calculated as the ratio of the absorbance intensity at *t* = 0, 1, 2, 3, 4, and 5 h to the absorbance intensity at *t* = 0 h. Based on these plots, it can be observed that as time progresses, the *C*/*C*_0_ decreases. The photocatalytic activity of 50% CdS@MoS_2_ against BG, MB, and RhB dyes was found to be substantially higher than those of pristine MoS_2_, CdS, 10% CdS@MoS_2_ and 30% CdS@MoS_2_.

The first-order rate constants for the degradation of BG, MB, and RhB dyes by 50% CdS@MoS_2_ were estimated to be 0.0145, 0.0087, and 0.0038 min^− 1^, respectively, as shown in Figure S8. The low photocatalytic degradation activity of MoS_2_ could be attributed to the rapid recombination of e^−^/h^+^ pairs. The superior photocatalytic activity of 50% CdS@MoS_2_ is due to the presence of CdS on the surface of MoS_2_ which may facilitate the separation and transfer of the photogenerated electrons and holes effectively, hence, better photocatalytic degradation efficiency. The improved photocatalytic activity of 50% CdS@MoS_2_ may also be due to its high surface area (63.542 m^2^/g) and close interfacial contact, which enables the dye molecules to adsorb onto the photocatalyst (adsorption is a prerequisite for photocatalytic reactions [33]) and able to degrade these dyes upon light irradiation.

Based on these results, it is also observed that 50% CdS@MoS_2_ could degrade more BG than MB and RhB dye. In general, photocatalytic degradation is strongly related to the adsorption affinity of dyes on the surface of the photocatalyst [33]. This indicates that BG dye has a higher affinity towards 50% CdS@MoS_2_ when compared to MB and RhB under the same conditions. This can be evident during the adsorption-desorption equilibrium at 0 h in which about 34.7%, 23.8%, and 16.5% of BG, MB, and RhB could be adsorbed on 50% CdS@MoS_2_, respectively. Thus, high adsorption leads to more interaction between dyes with the active sites of the 50% CdS@MoS_2_, resulting in better photocatalytic degradation. Table 2 compares photocatalytic activity of the synthesized CdS@MoS_2_ with previously reported work. Amongst them, the hydrothermally synthesized CdS@MoS_2_ reported by Liu and co-workers exhibited nearly the same activity [20]. However, in their study, they used a higher wattage of light (500 W) and only one type of dye (MB) was studied. On the other hand, in the present study, three different dyes were used to highlight the selectivity and efficiency of the synthesized CdS@MoS_2_ with a minimal dosage of catalyst (0.1 mg/mL) under irradiation by a lower wattage light source (300 W). Moreover, unlike these previous studies, here we report an in-depth study of the active species responsible for dye degradation through suitable trapping experiments to better understand the underlying mechanisms driving the degradation of BG, MB, and RhB dyes.

**Table 2:** Previous studies on the photocatalytic degradation of dyes using CdS/MoS_2_-based composites

Synthesized materials	Source of light	Pollutant used	Amount of photocatalyst used	Rate constant (min^− 1^)	Results	Ref.
ZnS/Mn-CdS/ MoS_2_/TiO_2_	300 W of Xe lamp	10 ppm of methyl orange (100 mL)	20 mg (0.2 mg/mL)	Not mentioned	98% in 100 min	[49]
MoS_2_/CdS/ Bi_2_MoO_6_	300 W Xe lamp	10 ppm of rhodamine B, methyl orange, methylene blue, and bromophenol blue (50 mL)	30 mg (0.6 mg/mL)	0.0256	Rhodamine B: 70.5%, Methyl orange:80.3%, Methylene blue: 96.0%, and bromophenol blue: 99.1% in 120 min	[50]
CdS@MoS_2_	500 W Xe lamp	20 ppm of methylene blue (50 mL)	4 mg (0.08 mg/mL)	0.105	80% in 30 min	[20]
CdS@MoS_2_	300 W Xe lamp	10 ppm of brilliant green, methylene blue, and rhodamine B (50 mL)	5 mg (0.1 mg/mL)	Brilliant green: 0.0145 Methylene blue: 0.0087 and Rhodamine B: 0.0038	Brilliant green: 97.6% Methylene blue: 90.3%, and Rhodamine B: 75.5% in 5 h	This work

### Determination of the main reactive species with different scavengers

**Fig. 7:**
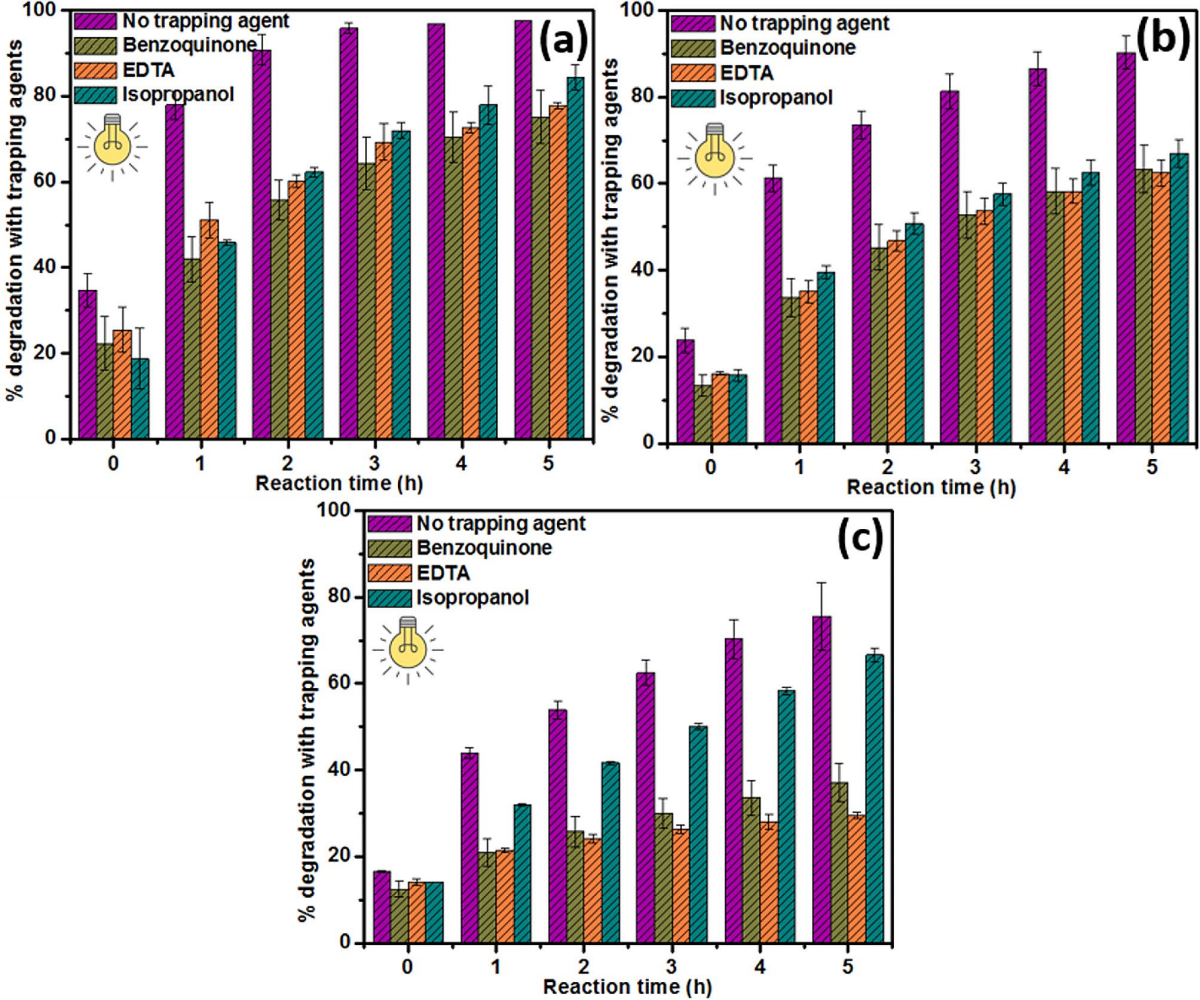
Photocatalytic activity of 50% CdS@MoS_2_ with different trapping agents to determine the main reactive species responsible for the degradation of (**a**) BG, (**b**) MB, and (**c**) RhB dye

Active species trapping experiments were performed to get deeper insight into the primary and secondary species responsible for the degradation of BG, MB, and RhB dyes during the photocatalysis process using 50% CdS@MoS_2_ composite. Different trapping agents (scavengers) including isopropanol, benzoquinone, and EDTA-2Na were used in separate experiments as scavengers of ^•^OH, O_2_^•−^, and h^+^, respectively, in the photocatalytic degradation process of the different dyes [40, 41]. Higher inhibition (lower percentage degradation) in the presence of a trapping agent implies higher importance of the corresponding active species in the degradation process. It was observed that when no scavenger was used, the degradation efficiency of 50% CdS@MoS_2_ was 97.6% ± 1.74%, 90.3% ± 3.80%, and 75.5% ± 7.78% for BG, MB, and RhB, respectively (Fig. 7(a − c)). As depicted in Fig. 7(a) for 50% CdS@MoS_2_, the presence of isopropanol inhibits BG degradation, resulting in a drop in degradation efficiency to 84.4% ± 3.02%. After the addition of benzoquinone and EDTA-2Na, the photocatalytic efficiency of 50% CdS@MoS_2_ decreased to 75.2% ± 6.20% and 77.7% ± 0.79%, respectively. Similarly, for the degradation of MB as shown in Fig. 7(b), both benzoquinone and EDTA-2Na can inhibit the photocatalytic efficiency of MB degradation by 50% CdS@MoS_2_ from 90.3% ± 3.80–63.3% ± 5.51% and 62.5% ± 3.06%, respectively. Moreover, in the presence of isopropanol, 50% CdS@ MoS_2_ could degrade about 67.0% ± 3.10% of MB. Based on these inhibitions, it can be observed that the difference between trapping agents was less pronounced for BG and MB dyes, but on average isopropanol was still less inhibiting than EDTA-2Na and benzoquinone. However, it is different in the case of RhB degradation. As shown in Fig. 7(c), the photocatalytic efficiency of RhB degradation by 50% CdS@MoS_2_ decreased from 75.5% ± 7.78% in the absence of scavenger to 66.6% ± 1.70% in the presence of isopropanol. In comparison, the photocatalytic RhB degradation efficiency decreased to 37.1% ± 4.33% and 29.5% ± 0.75% after the addition of benzoquinone and EDTA-2Na, respectively. Based on the slight influence of isopropanol on the degradation of RhB, it can be concluded that ^•^OH was not the main active species during the photocatalytic degradation of RhB. Contrarily, the obvious inhibition of benzoquinone and EDTA-2Na in the RhB degradation illustrates that h^+^ and O_2_^•−^ were the main reactive species during the photocatalytic dye degradation process. Overall, the various active species contribute in the order h^+^ > O_2_^•−^ > ^•^OH for the degradation of RhB. Thus, it can be inferred that h^+^, O_2_^•−^, and ^•^OH are all generated in the photocatalytic process using 50% CdS@MoS_2_. Additionally, the synergistic effect between the different active species can greatly enhance the photocatalytic performance.

### Possible photocatalytic mechanism for dye degradation

**Fig. 8:**
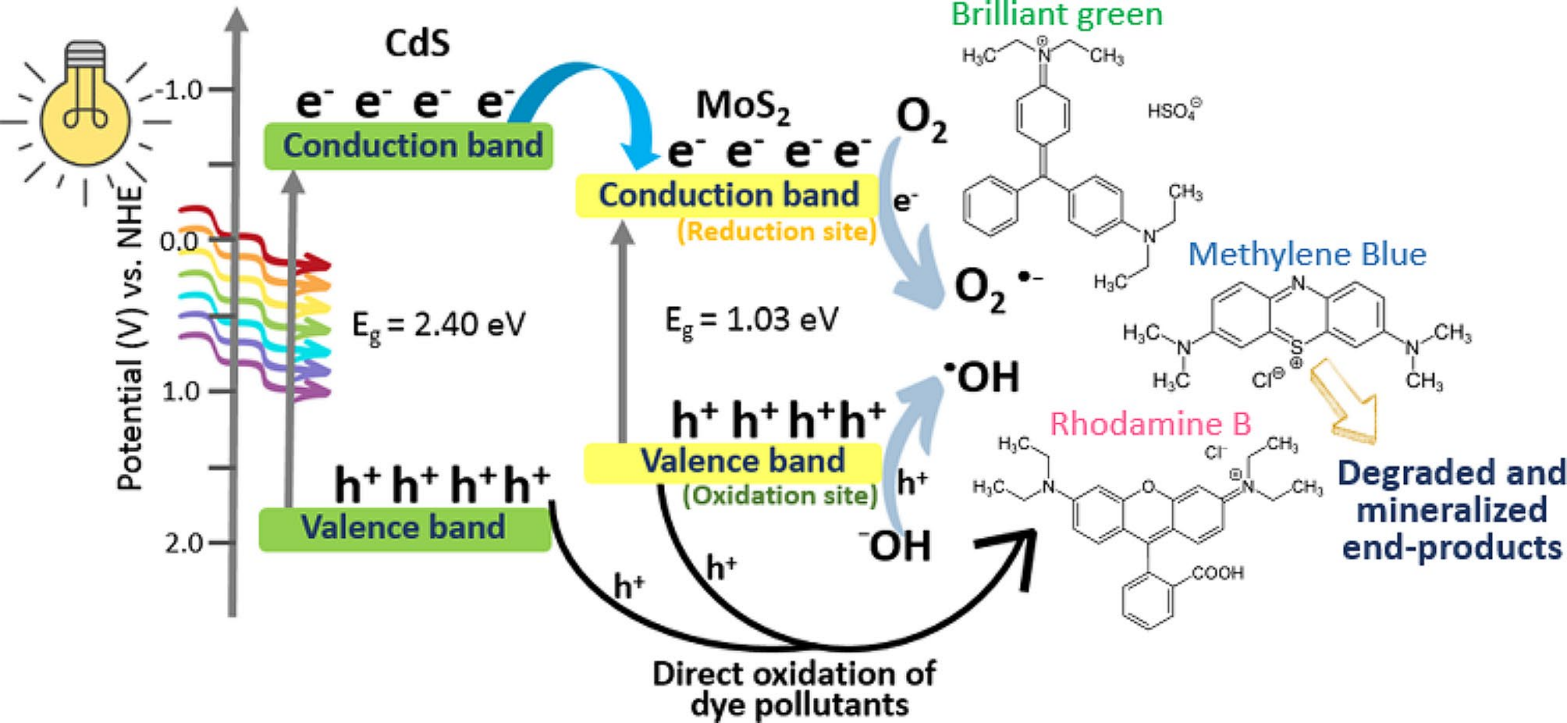
Proposed mechanism for the photocatalytic degradation of different dyes using CdS@MoS_2_

Based on the above results, the mechanism for photocatalytic degradation of different dyes using CdS@MoS_2_ was proposed and is shown in Fig. 8. Based on the literature, MoS_2_ is assumed to have *E*_CB_ = 0.04 V and *E*_VB_ = 1.36 V, while CdS has *E*_CB_ = -0.63 V and *E*_VB_ = 1.77 V [46]. Upon visible light irradiation, CdS simultaneously generates e^−^ and h^+^, in which photogenerated e^−^ are excited to the CB, leaving behind h^+^ in the VB. Next, the photogenerated e^−^ migrates to the CB of MoS_2_ to take part in the reduction process. The effective charge separation contributes to the improved photocatalytic activity of CdS@MoS_2_. The photogenerated e^−^ can reduce the dye directly or react with electron acceptors adsorbed on the surface of the photocatalyst, such as dissolved O_2_ in water, which is reduced it to O_2_^•−^. On the other hand, the photogenerated h^+^ can react with H_2_O or OH^−^ species, oxidizing them into ^•^OH radicals, or directly oxidizing the organic dyes to CO_2_ and H_2_O, which are harmless end products [47, 48]. Based on the active species trapping study, both benzoquinone and EDTA-2Na showed the highest inhibitions when compared to no scavenger for all three dyes. However, there is no significant difference between the inhibition caused by benzoquinone and EDTA-2Na, which indicates that both O_2_^•−^ and h^+^ play equally important roles in the photocatalytic degradation process. Moreover, in the case of RhB degradation, isopropanol shows the lowest inhibition, implying the least importance of ^•^OH taking part in the RhB degradation.

## Conclusion

In this study, MoS_2_, CdS, 10% CdS@MoS_2_, 30% CdS@MoS_2_, and 50% CdS@MoS_2_ were successfully synthesized using a hydrothermal method. The XRD patterns of pristine MoS_2_ and CdS exhibited mixed-phase crystal structures, while the XRD patterns of CdS@MoS_2_ composites revealed both the presence of 3R/2H MoS_2_ and a mixed phase of hexagonal and cubic CdS. The formation of the synthesized MoS_2_, CdS, and CdS@MoS_2_ composites was also confirmed by FT-IR analysis. The effective optical band gap of MoS_2_ increases from 1.03 eV to 2.3 eV as the CdS content increases to 50%. The photocatalytic degradation activity of these materials was investigated under visible light irradiation. Based on the results, 50% CdS@MoS_2_ exhibited the highest photocatalytic activity for the degradation of BG, MB, and RhB dyes under visible light irradiation. The active species trapping study shows no significant difference between the trapping agents in the inhibition of BG and MB degradation. However, for RhB degradation, it can be concluded that h^+^ and O_2_^•−^ are the main active species involved in the photocatalytic process under visible light irradiation. A possible mechanism for the photocatalytic degradation of the dyes using CdS@MoS_2_ was also proposed. These findings are expected to assist in the design and construction of highly efficient photocatalysts for the large-scale degradation of dye effluents.

### Electronic supplementary material

Below is the link to the electronic supplementary material.

Supplementary Material 1

## Data Availability

No datasets were generated or analysed during the current study.
