# Supplementary material for: Effect of CdS loading on the properties and photocatalytic activity of MoS2 nanosheets
Source: BMC Chem. 2024 Jul 24;18(1):135. doi: 10.1186/s13065-024-01250-y (PMC11270851; doi:10.1186/s13065-024-01250-y)
Supplement: Supplementary file 1 — Supplementary Material 1 [file 13065_2024_1250_MOESM1_ESM.pdf]

Supplementary Material

Effect of CdS loading on the properties and photocatalytic activity of MoS_2 nanosheets

Ashmalina Rahman[1], Fazlurrahman Khan[2,3,4], James Robert Jennings[5,6], Ai Ling Tan[1], Young-Mog Kim[3,4,7], Mohammad Mansoob Khan[1,6]*

[1]Chemical Sciences, Faculty of Science, Universiti Brunei Darussalam, Jalan Tungku Link, Gadong, BE 1410, Brunei Darussalam.

[2]Institute of Fisheries Science, Pukyong National University. Busan 48513, Republic of Korea.

[3]Marine Integrated Biomedical Technology Center, The National Key Research Institutes in Universities, Pukyong National University, Busan 48513, Republic of Korea.

[4]Research Center for Marine Integrated Bionics Technology, Pukyong National University, Busan 48513, Republic of Korea.

[5]Applied Physics, Faculty of Science, Universiti Brunei Darussalam, Jalan Tungku Link, Gadong, BE 1410, Brunei Darussalam.

[6]Optoelectronic Device Research Group, Universiti Brunei Darussalam, Jalan Tungku Link, Gadong, BE 1410, Brunei Darussalam.

[7]Department of Food Science and Technology, Pukyong National University, Busan 48513, Republic of Korea.

*Email: mmansoobkhan@yahoo.com and mansoob.khan@ubd.edu.bn

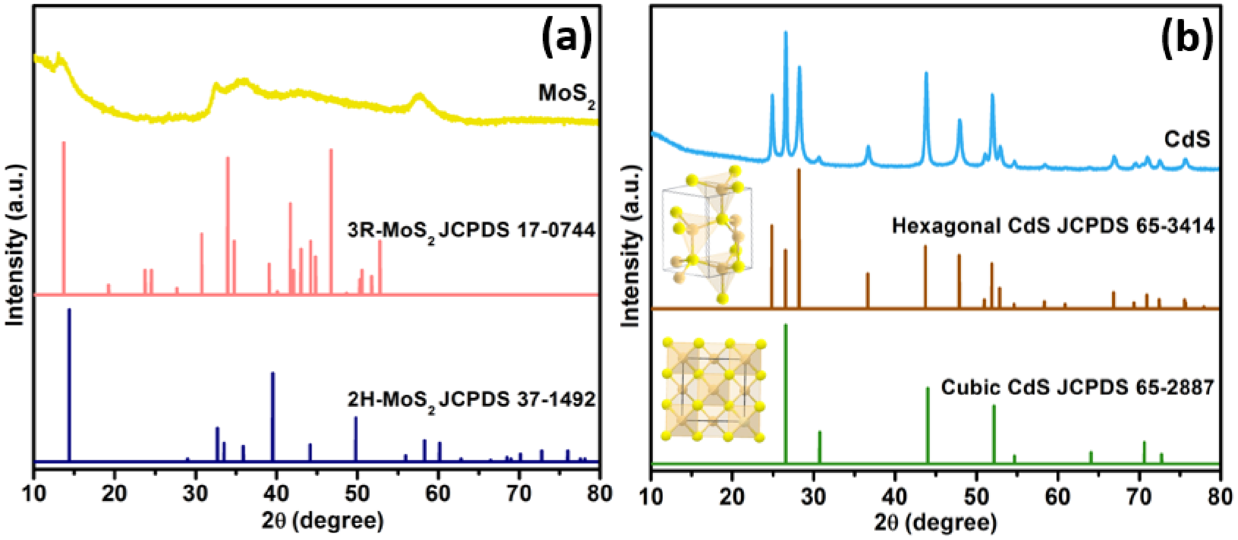

Figure S1. XRD patterns of **(a)** MoS_2 and **(b)** CdS with their respective standards.

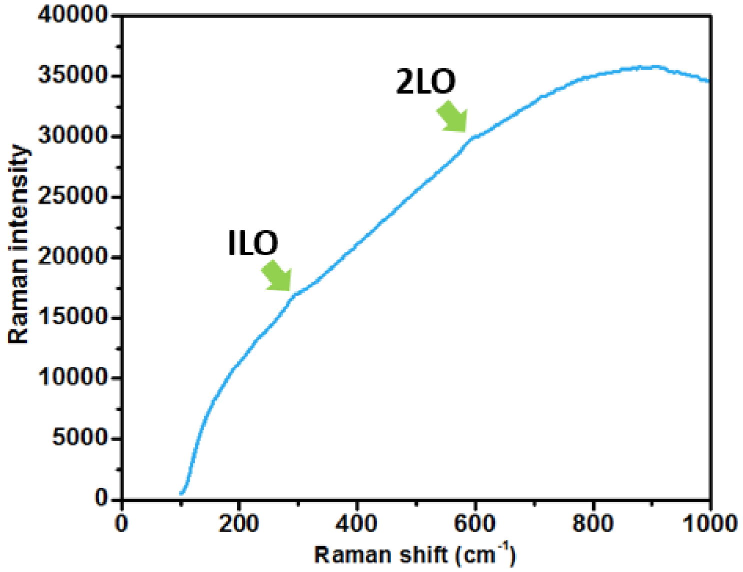

Figure S2. Raman spectrum of CdS.

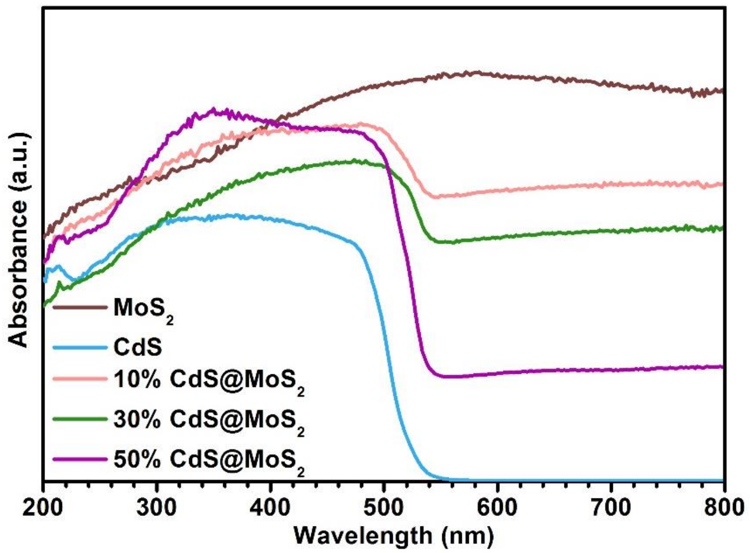

Figure S3. Absorbance (computed as the negative logarithm of the reflectance) spectra of MoS_2, CdS, 10% CdS@MoS_2, 30% CdS@MoS_2, and 50% CdS@MoS_2.

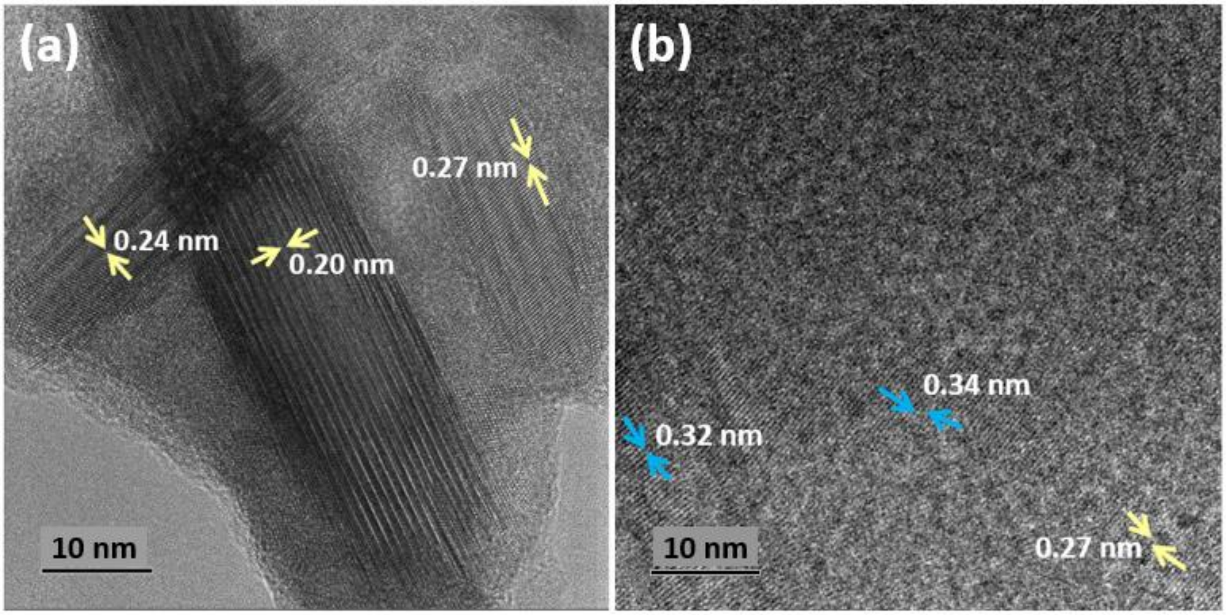

Figure S4. HR-TEM images of **(a)** MoS_2 and **(b)** 50% CdS@MoS_2 composite.

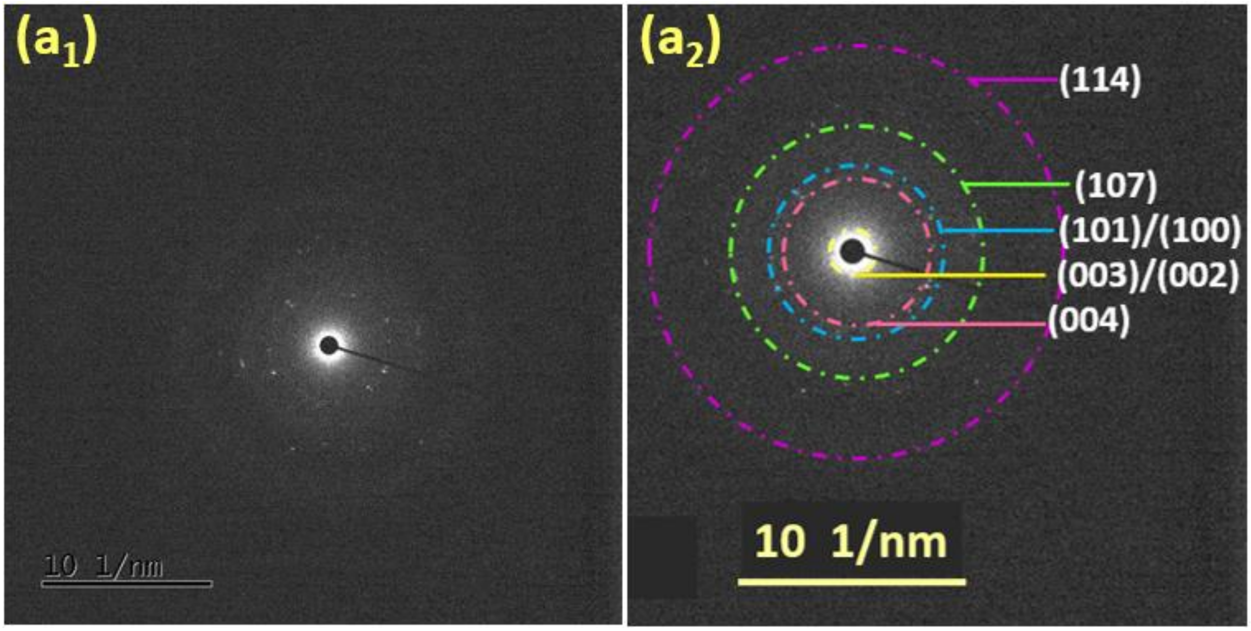

Figure S5. (a$_1$) Original and **(a$_2$)** indexed SAED patterns of MoS_2.

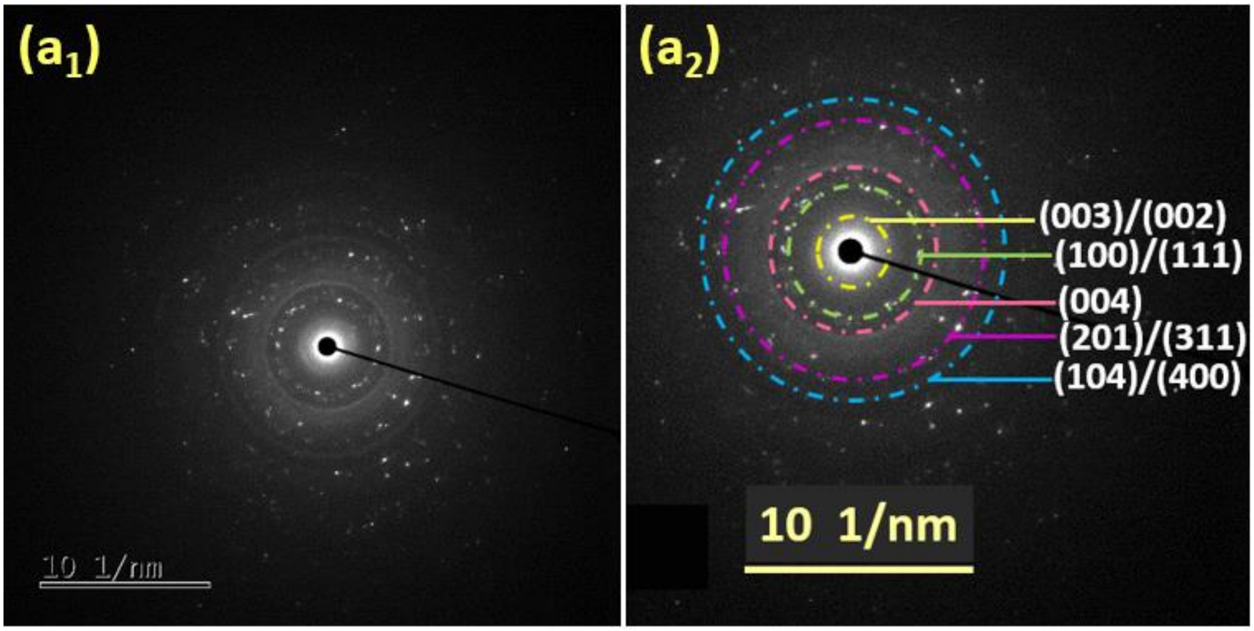

Figure S6. (a$_1$) Original and **(a$_2$)** indexed SAED patterns of 50% $CdS@MoS_2$.

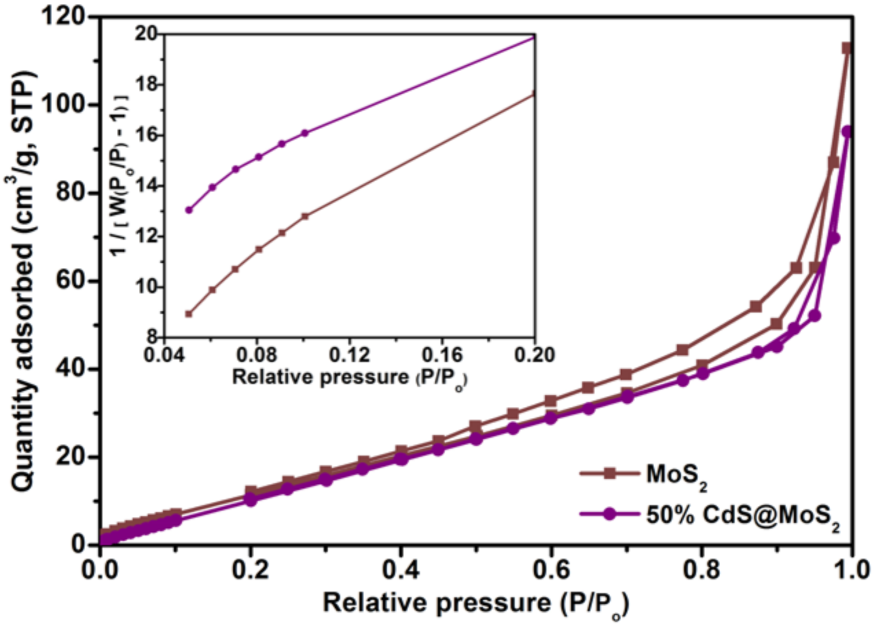

Figure S7. N_2 adsorption and desorption isotherms and multipoint BET plots (inset) of MoS_2 and 50% $CdS@MoS_2$.

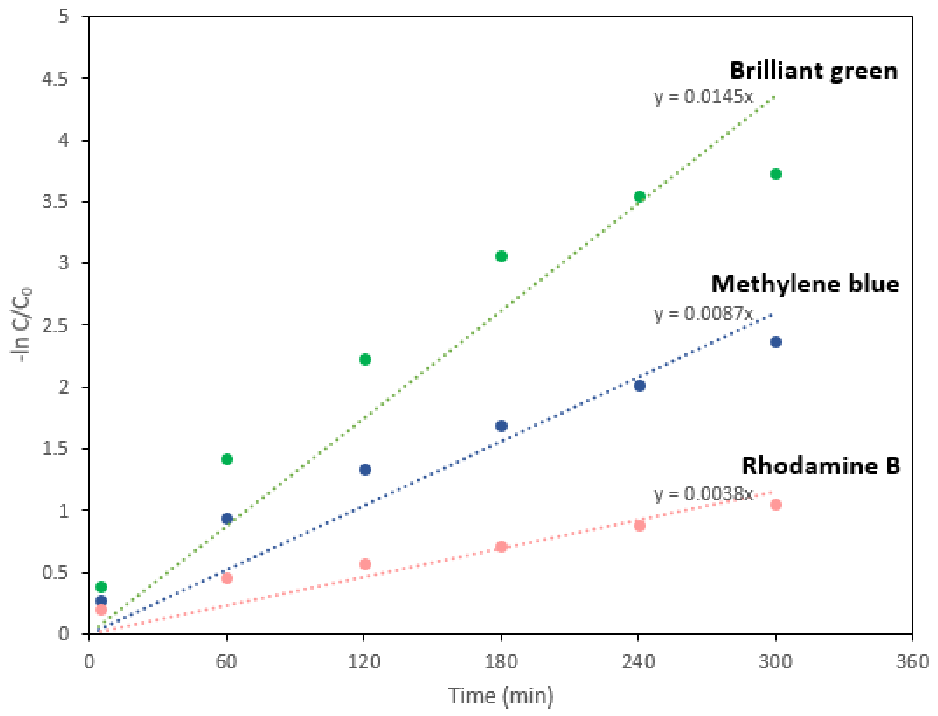

Figure S8. First order kinetic plots (-ln(C/C_0) vs. time) for the photocatalytic degradation of BG, MB, and RhB dyes using 50% CdS@MoS_2 under ~14 mW/cm^2 UV-visible ($\lambda > 350$ nm) light irradiation.
